# Outbreak strain characterisation and pharyngeal carriage detection following a protracted group B meningococcal outbreak in adolescents in South-West England

**DOI:** 10.1038/s41598-019-46483-3

**Published:** 2019-07-10

**Authors:** Stephen A. Clark, Jay Lucidarme, Georgina Angel, Aiswarya Lekshmi, Begonia Morales-Aza, Laura Willerton, Helen Campbell, Steve J. Gray, Shamez N. Ladhani, Mike Wade, Mary Ramsay, Julie Yates, Adam Finn, Ray Borrow

**Affiliations:** 1 Meningococcal Reference Unit, Public Health England, Manchester, UK; 2 Public Health England South West, Bristol, UK; 3 School of Cellular and Molecular Medicine, University of Bristol, Bristol, UK; 4 Immunisation and Countermeasures Division, Public Health England, London, UK

**Keywords:** Microbiology techniques, Policy and public health in microbiology, Peptide vaccines

## Abstract

Between April 2016 and September 2017, four cases of group B meningococcal disease were reported among sixth-form college students in Bristol, UK. Culture and non-culture whole genome sequencing was utilised and demonstrated that the four genomes of the responsible ST-41 strains clustered closely on a sub-lineage of ST-41/44 clonal complex. The outbreak resulted in two fatalities. A distinct social group associated with one of the cases was selected for vaccination with 4CMenB and pharyngeal swabbing. *In vitro* culturing, multiple real-time PCR assays (*sodC*, *ctrA* and *siaD*_*B*_) and a PorA PCR-sequencing assay were used to detect meningococcal colonisation and a carriage rate of 32.6% was observed. Furthermore, a high proportion of the pharyngeal swabs (78.3%) yielded a Factor H-Binding Protein (fHbp) nucleotide allele suggesting that the antigenic gene is prevalent among non-meningococcal flora, most likely *Neisseria* commensals. This may have implications for fHbp as a vaccine antigen should it be shown to influence bacterial colonisation.

## Introduction

Educational establishments have long been associated with increased rates of invasive meningococcal disease (IMD). A recent study suggested that, in England, the relative risk of IMD is 11 times higher (95% CI 4.7–28.7) among university students compared to non-students from the same age group (15–24 years)^1^. This pattern has largely been attributed to a relatively high carriage rate among adolescents, which can be driven by close quarter living arrangements (e.g. university accommodation), intimate social behaviours and/or movement of people from wider geographical areas; all of which can contribute to the transmission of hyper-virulent *Neisseria meningitidis* strains^2,3^.

In order to reduce group B (MenB) IMD in the UK, 4CMenB (Bexsero®, GlaxoSmithKline, Belgium) was introduced into the national infant immunisation schedule in late 2015 as a two-dose priming schedule at 8 and 16 weeks with a booster at 12 months. The vaccine has proven to be highly effective, with MenB cases reduced by nearly a half in the vaccine eligible infant cohort within 10 months of the introduction of the programme^4^. Another protein-based meningococcal vaccine, rLP2086 (Trumenba®, Pfizer, US), is also licenced for use in Europe for those aged from 10 years^5^. In the UK, the routine use of these vaccines in adolescents is unlikely to be cost effective without clear evidence of an impact on meningococcal carriage and the consequent induction of herd protection to unvaccinated age groups, especially infants and toddlers who have the highest incidence of MenB disease^6^. Nonetheless, both of these vaccines are available and have been used in response to MenB university outbreaks in the United States^7,8^.

Between April 2016 and September 2017, an outbreak of MenB disease occurred in adolescents associated with a sixth form college of 3000 students (16–18 years) in Bristol in South West England. Unlike universities, sixth form colleges do not typically have communal living arrangements and, in this case, students commuted in from a wide geographical area (across five different administrative counties). Here we describe the progression of the outbreak, the public health response, subsequent epidemiological investigations and the results of a pharyngeal swabbing exercise carried out to assess meningococcal carriage among an extended social group close to the outbreak.

## Results

### Initial cases: Identification and public health response

**Figure 1:**
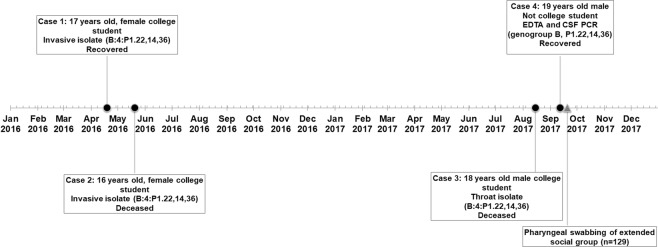
A timeline illustrating the timing and details of four adolescent cases during a MenB meningococcal outbreak in Bristol in 2016 and 2017. Two cases occurred in college students in early 2016 and a further two cases occurred in late 2017. A pharyngeal swabbing exercise was performed among an extended social group shortly after case 4.

The outbreak is summarised in Fig. 1. The first case (case 1) was in a 17 year old female student at a sixth form college in Bristol (henceforth referred to as ‘the college’). The patient was admitted in April 2016 with headache, fever, abdominal pain and a rash. A blood sample yielded a meningococcal isolate with phenotype B:4:P1.NT,14,NT (NT = non-typable using standard antibody panel). fHbp and PorA genotyping revealed a genosubtype of P1.22,14,36 and an *fHbp* allele encoding peptide 1.4. Whole genome sequencing allowed multi-locus sequencing typing (MLST) analysis and the isolate belonged to sequence type (ST) 41 (ST-41/44 complex). The patient recovered without complications.

Case 2, which occurred five weeks after case 1, was also in a student at the college. The patient (16 year old female) presented at hospital but was sent home with suspected viral illness. The patient returned the following day with photophobia and confusion and was admitted to the local intensive care unit. Her condition deteriorated over the following 24 hours and she died the next day.

Blood sampling yielded a meningococcal isolate with identical phenotype, ST and genotypic antigen profile as the strain from Case 1.

Following both cases 1 and 2, respective household contacts were offered antibiotic chemoprophylaxis in accordance with national guidelines^9^. Despite both cases yielding isolates with identical phenotypes, no shared friendship groups or school classes were identified. A detailed public health risk assessment concluded that mass chemoprophylaxis and/or vaccination in the college were unwarranted unless a further linked case occurred within the following three months. A plan for wider chemoprophylaxis and/or vaccination was suspended in preparation for such an eventuality. Both cases featured in the local and national media and campaigns raising awareness about the signs and symptoms of IMD were undertaken by the college and local health teams. No further epidemiologically-linked IMD cases were identified in 2016.

### Initial cases: Further characterisation

The two isolates were subsequently predicted to be covered by the fHbp component in 4CMenB using the Meningococcal Antigen Typing System (MATS, fHbp RPs = 0.043 and 0.050 for cases 1 and 2, respectively). None of the other 4CMenB antigens were predicted to be protective. The Meningococcal Antigen Surface Expression (MEASURE) assay was used to assess coverage of rLP2086. Mean Fluorescent Intensity (MFI) values for both isolates were above the putative coverage threshold of 1000 for rLP2086 (1,697 and 2,152 for cases 1 and 2, respectively).

### Later cases: Identification and public health response

Case 3 occurred in August 2017; 15 months after case 2. The patient (18 year old male) was also a student of the college and presented with vomiting, confusion, severe lower limb pain and a rash whilst attending a music festival. The patient went into cardiac arrest and was transferred to intensive care in a comatose state. The patient later died after life support was withdrawn. Clinical samples taken failed to yield an isolate. Initial laboratory diagnosis confirmation was achieved using PCR only (*ctrA* Taqman®) on a blood sample, however, subsequent pharyngeal swabbing of the patient yielded a meningococcal isolate with the same typing profile (B:4:P1.22,14,36) and fHbp peptide (1.4) as the two isolates from the initial cases. The patient was reported as being a friend of the students in the previous two cases. The case was widely reported in the media.

Case 4 occurred in a 19 year old male in early September 2017, one month after case 3. The patient was a former student at the college and lived nearby. In this case, the patient initially presented with vomiting, rigors, and drowsiness before being admitted to ICU and given antibiotic treatment. The patient recovered without sequelae. No meningococcal isolate was obtained from samples taken (blood and CSF) and laboratory confirmation was by PCR only. All clinical specimens were PCR positive for group B and non-culture fHbp and PorA molecular typing revealed profiles matching the other cases (fHbp 1.4 and PorA P1.22,14,36). All close contacts were offered antibiotic chemoprophylaxis according to national guidance.

### Later cases: Further characterisation

The throat isolate from case 3 and a blood sample from case 4 were whole genome sequenced. MLST analysis revealed the strains from cases 3 and 4 also belonged to ST-41. The isolate was covered by fHbp component in 4CMenB (fHbp RP: 0.039) and by rLP2086 (MEASURE MFI: 2357).

### Identification of social group eligible for chemoprophylaxis and vaccination

Following case 4, the outbreak control team recognised the patient as part of a wider social group, many of whom had attended the funeral of case 3, as well as other related social gatherings. Due to the links between the final two cases, it was agreed that this wider social group (primarily friends and family of case 3) would be offered chemoprophylaxis and vaccination. The eligibility criteria were based on attendance of the funeral of case 3 and/or associated social gatherings. A total of 202 people were invited to attend a vaccination clinic at the college. Of these, 138 people were given chemoprophylaxis and one dose of 4CMenB. 4CMenB was chosen due to the positive predicted strain coverage (based on the MATS) and stocks of the vaccine being available at the time.

### Genomic comparisons of case genomes

**Figure 2:**
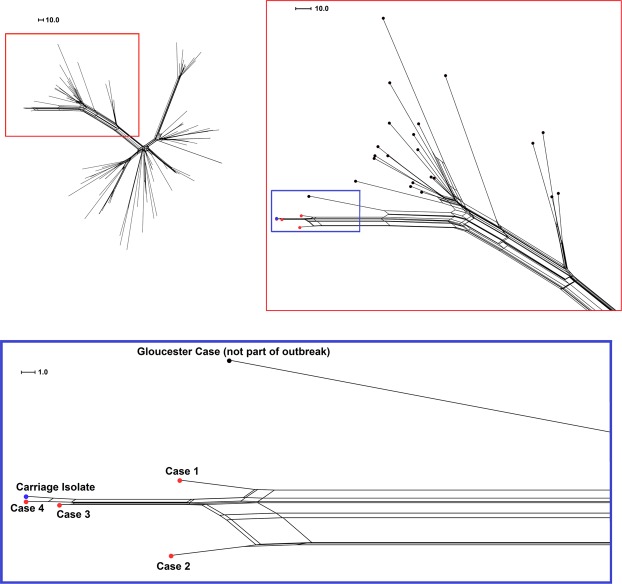
Neighbor-net phylogenetic network of outbreak strains among sub-lineage of the ST-41/44 complex. Genomic analyses were performed at 1546 loci and included a sub-lineage of ST-41/44 complex meningococcal genomes including the four case isolates and a carriage isolate (n = 102: UK = 100 and Ireland = 2). Top left: The network of all selected strains. Top right: zoomed section of network corresponding to area within the red box on opposite image. Below: Further zoomed section of network corresponding to the area within the blue box in the top right image. Outbreak strains, the carriage isolate (swab 5) and a closely-related strain from an unconnected disease case are labelled and highlighted in red, blue and black, respectively.

The Neighbor-Net phylogenetic network in Fig. 2 illustrates the genetic relatedness of outbreak strains within a subset of ST-41/44 complex strains that form a sub-lineage of the main clonal complex (n = 102). The four case genomes clustered closely as a distinct sub-cluster off one of the main branches of the network (Fig. 2, top right panel). All but one of the isolates in this branch were sub-type P1.22,14 (n = 26/27). Interestingly, the closest-related isolate to the outbreak (Fig. 2, bottom panel) was P1.7,30–5, an indication that a single locus can be misleading in informing outbreak management. This isolate was from a disease case occurring in 2015 in Gloucester, approximately 30 miles from Bristol. The genomes from the first two outbreak cases were more distinct than the later cases, with 29 loci differences between case 1 and 2 (comprising 18 differences in individual genes and three putative multi-gene recombination events), and only five individual loci differences between cases 3 and 4.

### Pharyngeal swabbing and carriage detection

Those attending the aforementioned vaccination clinic at the college were asked to take part in a nasopharygeal swabbing exercise prior to vaccination and prophylaxis. Of the 138 attending the clinic, 129 gave consent to be swabbed. A breakdown of the age and sex of the swabbed participants is shown in Table 1. The majority (59.7%) of participants were female. Ages ranged from 3 to 74 years, with 59.7% of participants aged 15–24 years.

**Table 1:** Age and sex breakdown of participants of the nasopharyngeal swabbing exercise.

Age group (years)	Sex		Total
	Female (%)	Male	
0–4	1 (100)	0	1
5–14	3 (23.1)	10	13
15–24	48 (62.3)	29	77
>25	25 (65.8)	13	38
**Total**	**77** (**59.7)**	**52**	**129**

**Figure 3:**
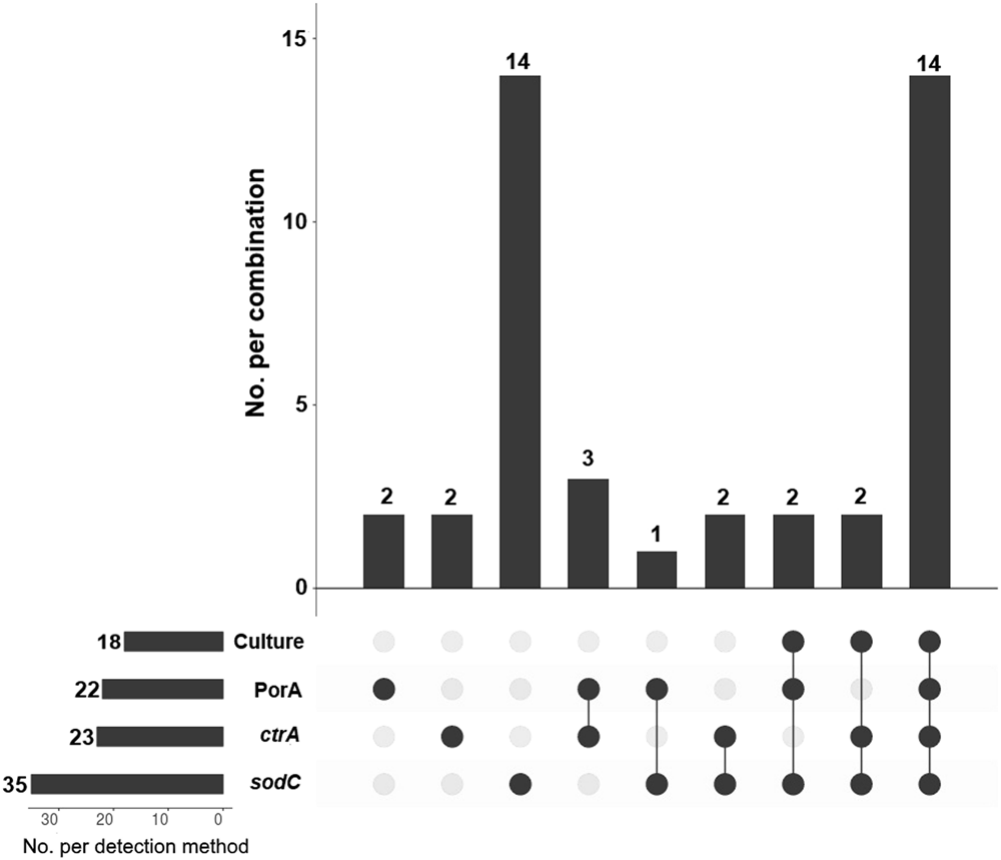
An UpSet plot of the numbers of pharyngeal swabs testing positive for meningococcal carriage by detection method. The bars and numbers to the bottom left represent the numbers of swabs positive for each detection method. The bars and numbers above represent the combinations of positive detection methods for all positive swabs as indicated below (n = 42). Combinations not represented by at least one swab were omitted.

Multiple laboratory methods were used to detect meningococcal carriage from the swabs (described in Methods). Figure 3 provides a breakdown of the positive results by detection method. Refer to Supplementary Table S1 for detailed carriage results. Overall, 42/129 (32.6%) of the swabs yielded at least one, but in many cases a combination of a meningococcal isolate, a positive *sodC* real-time PCR result, a positive *ctrA*/*siaD*_*B*_ real-time PCR result or a sequenced *porA* allele (Fig. 3).

Of the 129 swabs taken, 18 yielded an *N. meningitidis* isolate (Fig. 3, Supplementary Table S1). All but two of these isolates were obtained from participants within the 15–24 age group. One isolate (swab 5) was phenotypically indistinguishable from the clinical isolates obtained for cases 1–3 in the Bristol outbreak (B:4:P1.14) and core-genome MLST analysis showed that this isolate was clustered with all four genomes from the outbreak cases (Fig. 2). This isolate was closest to the strains from cases 3 and 4, differing at 6 and 4 loci, respectively. The carrying participant fell within the 15–24 age range.

Using a *sodC* real-time PCR assay, 96 of the 129 broths produced a positive amplification signal after 50 cycles. Of these, 35 samples produced Ct values below the established positive threshold of Ct 36 (Fig. 3, Supplementary Table S1). All 18 of the swabs from which *N. meningitidis* was isolated produced a positive *sodC* Ct value (<36).

Twenty three of the swabs produced a positive *ctrA* result (Ct ≤45) (Fig. 3, Supplementary Table S1). Two swabs yielding *N. meningitidis* isolates failed to produce a positive *ctrA* signal. Both of these isolates were phenotypically non-groupable and featured complete or partial deletions within the capsular locus (including deletion of *ctrA*).

Seventeen of the swabs that were positive for *sodC* were found to be negative for *ctrA* and/or *siaD*_*B*_. Five of the swabs that were negative for *sodC* were positive for *ctrA* (*ctrA* Ct values ranging from 36.7 to 39.2, *sodC* Ct values from 38.1 to 42.5 plus one swab undetected). A further two *sodC*-negative swabs produced a sequencable PorA product but no *ctrA* or *siaD*_*B*_ signal.

Using the PorA PCR-sequencing assay, 22/129 swabs (17.1%) produced a sequencable *porA* PCR product (Fig. 3, Supplementary Table S1). Sixteen of these 22 swabs also yielded a meningococcal isolate and a positive *sodC* PCR result. Of the remaining six samples, three were positive for *ctrA* but not *sodC* (Ct >36), one was positive for *sodC* but not *ctrA* and the remaining two PorA-sequenced swabs were negative for both *sodC* and *ctrA*. All PorA results obtained directly from the swabs matched those from the corresponding isolate genomes (Supplementary Table S1).

### Factor H Binding Protein sequencing

As an additional gene target, all swabs were tested using an fHbp PCR-sequencing assay. The fHbp results can be found in Supplementary Table S2 online. Unlike PorA, the *fHbp* traces in many cases featured a mixed signal and double electropherogram peaks. In total, 101/129 (78.3%) of the swabs yielded an *fHbp* PCR product, including 65/87 (74.7%) of the swabs that were negative by all other methods (Supplementary Table S2). For five of the 18 swabs that yielded isolates, there was a clear discrepancy between the *fHbp* allele sequenced directly from the swab and the *fHbp* allele of the corresponding isolate. For a further two, a double signal was seen in the traces, one of which was the *fHbp* allele harboured by the isolate (Supplementary Table S2). Overall, 15/101 (14.9%) of the sequenced *fHbp* alleles were ‘new’ alleles without an assigned PubMLST ID at the time of sequencing.

## Discussion

Between April 2016 and September 2017, a meningococcal outbreak occurred amongst sixth-form college students in Bristol, South-West England. Initial phenotypic characterisation suggested that the causative group B meningococci were closely related, and this was later confirmed by whole genome sequencing. Whole genome sequencing has become an almost indispensible tool for characterisation of endemic and outbreak strains. In most clusters/outbreaks, cases occur within a few days or weeks of each other and so the epidemiological links are often clear. However, outbreak cases occurring over a protracted timeframe can be more difficult to link and the genomic data from the invasive meningococcal strains can provide that definitive connection required to justify further public health action. To our knowledge, the outbreak detailed here was the first application of high-resolution genomic comparisons of both culture and non-culture genomes following a meningococcal outbreak. Whilst currently costly and not possible on all clinical samples, non-culture whole genome sequencing is likely to be used more and more given the high number of cases which do not yield a culturable isolate^10,11^.

In this outbreak, the causative organism was an ST-41 strain and the four meningococcal genomes recovered clustered very closely together phylogenetically within a distinct sub-lineage of the ST-41/44 complex. In recent years, the ST-41/44 complex has been the most common invasive MenB clonal complex in the UK and Europe, with most CC41/44 disease occurring in younger adults and children (<25 years old)^12,13^. The protracted nature of this outbreak was not dissimilar to MenB outbreaks observed in US universities in recent years, in particular the 2013 outbreak at a New Jersey university in which an ST-41/44 complex strain was responsible for nine cases over a 12 month period^7^. In that outbreak, the pre-licensure use of 4CMenB was authorised and over 5000 students were vaccinated. No further cases due to this strain were reported in the vaccinated cohort.

In this outbreak, the first two cases occurred in students attending the sixth form college during term-time. A lack of demonstrable contact or shared social groups between these two cases led to the decision not to undertake mass chemoprophylaxis or vaccination of the college. Cases 3 and 4 occurred during the following summer break, at least four weeks after the end of the college term. It is, therefore, likely that transmission of the strain was not restricted to the college setting and was occurring among friends and/or family outside the college. Interestingly, despite both occurring within the college, the genomes from the first two cases were more distinct than the latter two cases. The former cases were not closely acquainted but cases 3 and 4 were reportedly friends and part of the same social group. Thus, the relative similarity of the genomes in the latter cases may reflect this familiarity between the patients and possibly indicate a shorter transmission chain.

Following the fourth case, a high-risk social group identified by the outbreak control team took part in a swabbing exercise before being vaccinated with 4CMenB and chemoprophylaxed. One of the nasopharygeal isolates was very closely related to the strain responsible for the final case, with only four loci differences. This isolate was carried by an individual of student age, indicating ongoing transmission of this strain among this young social group which justifies the public health action taken.

Among the high-risk social group, an overall meningococcal carriage rate of 32.6% was observed using four detection methods and the results demonstrate the added value of using multiple detection methods in carriage studies. Two commonly-used real-time PCR targets, *sodC* and *ctrA* were utilised for carriage detection. The *sodC* Taqman® assay targets the gene encoding the Cu/Zn superoxide dismutase enzyme, which is well conserved among *N. meningitidis* strains^14^. The *ctrA* Taqman® assay targets a gene of the capsular locus which is highly conserved among capsulated invasive strains but often absent among non-capsulated commensal meningococci found in the oropharynx. Overall, seventeen of the swabs that were positive using the *sodC* assay were negative on the *ctrA* platform. This is not surprising given that many of the meningococci detected in the throat are likely to lack capsular genes. Interestingly, seven swabs that yielded a positive *ctrA, siaD*_*B*_ and/or PorA result, were negative in the *sodC* assay and five of these produced a *sodC* signal above the positive threshold of Ct 36 (up to Ct 42). One explanation is that at least some of these discrepancies are due to false *ctrA* positives, however, PorA was sequenced from three of the five *ctrA-*positive discrepant swabs. In addition, the *ctrA* assay has been used by Public Health England’s Meningococcal Reference Unit (PHE MRU) for clinical laboratory confirmation for ~20 years and, despite utilising a higher positive threshold (Ct 45), it shows a strong correlation with clinical IMD symptoms, strain isolation and successful genotyping directly from positive samples (including those with Ct values up to 45). Consequently, we suggest these results are likely to be false *sodC* negatives and, although the *sodC* assay is the most appropriate for carriage detection, the current positive threshold (Ct 36) may be too stringent. Any change in positivity thresholds can of course impact on assay specificity, so impact assessments/re-validation would be required prior to any change. One *ctrA/*PorA-positive and another PorA-positive swab were completely undetected by the *sodC* assay (after 50 cycles). The reasons for this are yet unclear but may indicate the presence of meningococcal strains without the *sodC* gene or with mutations that hinder PCR detection.

PorA and fHbp PCR-sequencing assays were also applied to the swabs to provide further characterisation. PorA PCR-sequencing provided additional detection and strain characterisation but, as expected, this assay format was not as sensitive as real-time PCR. A PorA-targeting real-time PCR assay could prove effective for carriage detection in future studies. Interestingly, amplification of *fHbp* PCR products did not correlate with positivity of the swabs using the other methods and a number of discrepancies were observed between *fHbp* from the swabs and the corresponding isolates. The fHbp assay was originally designed to amplify *fHbp* from meningococcal-confirmed clinical specimens, therefore, specificity was not crucial^15^. Indeed, amplification of NLA18150, a gene found in place of *fHbp* at the same locus in *N. lactamica*, was demonstrated among a small panel of *N. lactamica* isolates as part of the assay validation^15^. In 2013, Muzzi and colleagues identified *fHbp* in *N. cinerea, N. gonorrhoeae* and *N. polysaccharea*^16^. More recently, Lavender and colleagues demonstrated surface expression of functional fHbp by *N. cinerea* and its importance in down-regulating complement activity^17^. We, therefore, hypothesise that these mixed and unforeseen fHbp results are likely to be due to amplification of *fHbp* among both *N. meningitidis* and additional *Neisseria* species carried by the vast majority of the swabbed individuals. Whilst expression of fHbp by these hypothetical commensals could not be demonstrated here, and the impact of fHbp-containing vaccines on pharyngeal carriage is still in doubt, questions may be raised regarding the possible impact of these vaccines on non-meningococcal flora.

In conclusion, this manuscript describes the public health investigation and intervention following a MenB college outbreak and details the carriage profile of individuals close to the cases. Whole genome sequencing was crucial in linking the protracted cases. In addition to chemoprophylaxis of close contacts, the public health team carried out chemoprophylaxis and vaccination of a high risk social group which was later shown to be harbouring the outbreak strain. The pharyngeal swabbing data illustrate the benefit of using multiple laboratory techniques/genetic targets to detect meningococcal carriage and the surprisingly high prevalence of *fHbp* suggests that the gene is harboured by a wider proportion of the nasopharygeal flora than previously recognised.

## Methods

### Identification of cases and public health management

All suspected IMD disease cases were initially reported to the local PHE Health Protection Team (HPT). The HPT compiled relevant clinical and social information, supported local responders and coordinated outbreak response activities.

### Phenotypic characterisation of *N. meningitidis* isolates

Meningococcal case isolates were grown from blood or CSF samples by the local microbiology laboratory. Suspected meningococcal isolates were transferred to PHE MRU for species confirmation and characterisation.

Species confirmation of all (carriage and case) isolates was performed using oxidase, Gram stain and/or carbohydrate utilisation tests. All isolates were initially characterised phenotypically (serogroup, serotype, serosubtype) using dot-blot ELISA or co-agglutination^18^.

For invasive disease isolates, the MATS and the MEASURE assay were used to predict protective coverage of 4CMenB and rLP2086, respectively^19,20^. In the former, the relative potency (RP) of the recombinant 4CMenB antigens (fHbp, NHBA, NadA) was measured alongside PorA VR2 characterisation. Strains exhibiting RPs above defined positive bactericidal thresholds (PBT) and/or possessing the PorA P1.4 epitope were predicted to be covered by 4CMenB^21^. In the MEASURE assay, the MFI readout is correlated with fHbp surface expression. Strains producing MFI values of >1000 are predicted to be covered by rLP2086^20^.

### IMD confirmation using real-time PCR

In all cases, clinical specimens were sent to PHE MRU for nucleotide detection using a *ctrA*-targeting Taqman® assay. This screening assay also consists of primers and probes targeting the *siaD*_*B*_ (*csb*) gene allowing capsular group B detection^22^.

### Genotypic strain characterisation

PCR-sequencing assays targeting *fHbp* and *porA* were used to characterise these antigens from DNA extracted from isolates and non-culture specimens^15,23^. Carriage and clinical isolates underwent DNA extraction using the Wizard® Genomic DNA Purification Kit (Promega, US) and whole genome sequencing using the Ultra DNA Sample Prep Kit (NEBNext) in conjunction with the Illumina HiSeq. 2000 platform (100 bp paired-end reads). Whole genome sequencing of residual meningococcal DNA within clinical specimens (Case 4 only) was performed by University College London using the Agilent SureSelectXT system^11^. All genomic data were uploaded to the PubMLST database for annotation and indexing^24^.

Genomic comparisons were performed at 1,546 core genome loci using the PubMLST Genome Comparator tool^25^. All invasive ST-41/44 complex genomes within the PubMLST *Neisseria* database (n = 992) were randomly split into two groups (to improve processing speed) and each group underwent genomic comparison along with the outbreak isolates and carriage isolate from swab 5. The resultant distance matrices were visualised as Neighbor-net phylogenetic networks using Splitstree 4^26^. For each group, genomes that formed a sub-lineage of the clonal complex containing the outbreak/carriage strains of interest (total n = 102) were selected. The genomic analysis was then repeated with these 102 strains only.

### Oropharyngeal swabbing

As part of the public health intervention, a social group linked to cases 3 and 4 was identified for vaccination and chemoprophylaxis. In total, 129 provided informed consent to have oropharyngeal swabs collected. The swabs were suspended in 1.5 mL of skim milk-tryptone-glucose-glycerol (STGG) broth and stored at −70 or −80 °C. Pharyngeal swabbing of volunteers was performed as part of public health action in accordance with Public Health England guidelines therefore ethical approval was not required. Informed consent was obtained from all volunteers prior to vaccination and pharyngeal swabbing.

### Carriage detection: culture method

Upon arrival in the laboratory, the broths were vortexed and 100 µL was plated on to selective Gonococcal (GC) agar plates with VCAT (Vancomycin, Colistin, Amphotericin B & Trimethoprim) supplement (Oxoid, UK). Plates were incubated for up to 72 hours at 37 °C with 5% CO_2_. Suspected meningococcal colonies were sub-cultured on to Columbia horse blood agar (Oxoid, UK) followed by overnight incubation. All carriage isolates were characterised as stated previously.

### Carriage detection: PCR methods

Multiple molecular detection methods were utilised in order to detect meningococcal carriage. Firstly, a real-time PCR assay targeting the *sodC* gene (Cu-Zn superoxide dismutase) was used^14,27^. DNA was extracted from the STGG broth using an automated QIAsymphony SP instrument and using QIAsymphony DSP Virus/Pathogen Mini Kit (version 1. Qiagen, UK) following the manufacturer’s instructions. A volume of 340 µL of STGG broth was used in the extraction and the DNA was eluted in 110 µL of elution buffer. Extracts producing a cycle threshold (Ct) value of >36 were deemed negative. The assay featured 50 cycles in total.

In order to improve detection and to determine the capsular status of the carried meningococci (where possible), further real-time PCR assays targeting the capsular polysaccharide genes *ctrA* and *siaD*_*B*_ were used^22^. In this case, DNA was extracted using the Biorobot MDx platform (Qiagen, UK) using 300 µL of STGG broth and eluting in 80 µL of elution buffer. Additionally, nested PorA and fHbp PCR-sequencing assays were used on the swab extracts to provide further detection power and genotypic characterisation^15,23^. For swabs producing *fHbp* alleles different from the corresponding isolate, the swabs were re-extracted and re-sequenced to confirm. Positive results were broken down by detection method and illustrated using UpsetR^28^.

## Supplementary information

Dataset 1

## References

[1] Mandal S (2017). Risk of invasive meningococcal disease in university students in England and optimal strategies for protection using MenACWY vaccine. Vaccine.

[2] MacLennan J (2006). Social behavior and meningococcal carriage in British teenagers. Emerg. Infect. Dis..

[3] Christensen H, May M, Bowen L, Hickman M, Trotter CL (2010). Meningococcal carriage by age: a systematic review and meta-analysis. Lancet Infect. Dis..

[4] Parikh SR (2016). Effectiveness and impact of a reduced infant schedule of 4CMenB vaccine against group B meningococcal disease in England: a national observational cohort study. Lancet.

[5] Perez JL (2018). From research to licensure and beyond: clinical development of MenB-FHbp, a broadly protective meningococcal B vaccine. Expert Rev. Vaccines.

[6] Christensen H, Trotter CL, Hickman M, Edmunds WJ (2014). Re-evaluating cost effectiveness of universal meningitis vaccination (Bexsero) in England: modelling study. BMJ.

[7] McNamara LA (2015). First Use of a Serogroup B Meningococcal Vaccine in the US in Response to a University Outbreak. Pediatrics.

[8] Soeters HM (2017). Meningococcal Carriage Evaluation in Response to a Serogroup B Meningococcal Disease Outbreak and Mass Vaccination Campaign at a College—Rhode Island, 2015–2016. Clin. Infect. Dis..

[9] Public Health England*. Guidance for the public health management of meningococcal disease in the UK* (2018).

[10] Heinsbroek E (2013). Added value of PCR-testing for confirmation of invasive meningococcal disease in England. J. Infect..

[11] Clark SA, Doyle R, Lucidarme J, Borrow R, Breuer J (2017). Targeted DNA enrichment and whole genome sequencing of *Neisseria meningitidis* directly from clinical specimens. Int. J. Med. Microbiol..

[12] Brehony C (2014). Implications of differential age distribution of disease-associated meningococcal lineages for vaccine development. Clin. Vaccine Immunol..

[13] Hill DMC (2015). Genomic epidemiology of age-associated meningococcal lineages in national surveillance: an observational cohort study. Lancet Infect. Dis..

[14] Thomas JD (2011). SodC-based real-time PCR for detection of *Neisseria meningitidis*. PLoS One.

[15] Clark SA, Lucidarme J, Newbold LS, Borrow R (2014). Genotypic Analysis of Meningococcal Factor H-Binding Protein from Non-Culture Clinical Specimens. PLoS One.

[16] Muzzi A, Mora M, Pizza M, Rappuoli R, Donati C (2013). Conservation of Meningococcal Antigens in the Genus *Neisseria*. MBio.

[17] Lavender H, Poncin K, Tang CM (2017). *Neisseria cinerea* Expresses a Functional Factor H Binding Protein Which Is Recognized by Immune Responses Elicited by Meningococcal Vaccines. Infect. Immun..

[18] Gray SJ (2006). Epidemiology of meningococcal disease in England and Wales 1993/94 to 2003/04: contribution and experiences of the Meningococcal Reference Unit. J. Med. Microbiol..

[19] Donnelly J (2010). Qualitative and quantitative assessment of meningococcal antigens to evaluate the potential strain coverage of protein-based vaccines. Proc. Natl. Acad. Sci..

[20] McNeil, L. K. *et al*. Predicting the Susceptibility of Meningococcal Serogroup B Isolates to Bactericidal Antibodies Elicited by Bivalent rLP2086, a Novel Prophylactic Vaccine. *MBio* **9** (2018). 10.1128/mBio.00036-18 PMC5850321 29535195

[21] Parikh Sydel R, Newbold Lynne, Slater Stephanie, Stella Maria, Moschioni Monica, Lucidarme Jay, De Paola Rosita, Giuliani Maria, Serino Laura, Gray Stephen J, Clark Stephen A, Findlow Jamie, Pizza Mariagrazia, Ramsay Mary E, Ladhani Shamez N, Borrow Ray (2017). Meningococcal serogroup B strain coverage of the multicomponent 4CMenB vaccine with corresponding regional distribution and clinical characteristics in England, Wales, and Northern Ireland, 2007–08 and 2014–15: a qualitative and quantitative assessment. The Lancet Infectious Diseases.

[22] McHugh MP, Gray SJ, Kaczmarski EB, Guiver M (2015). Reduced turnaround time and improved diagnosis of invasive serogroup B *Neisseria meningitidis* and *Streptococcus pneumoniae* infections using a lyophilized quadruplex quantitative PCR. J. Med. Microbiol..

[23] Urwin, R. In *Methods in molecular medicine* **67**, 157–172 (2001). 10.1385/1-59259-149-3:157 21337144

[24] Jolley KA, Maiden MCJ (2010). BIGSdb: Scalable analysis of bacterial genome variation at the population level. BMC Bioinformatics.

[25] Bratcher HB, Corton C, Jolley KA, Parkhill J, Maiden MCJ (2014). A gene-by-gene population genomics platform: *de novo* assembly, annotation and genealogical analysis of 108 representative *Neisseria meningitidis* genomes. BMC Genomics.

[26] Huson DH, Bryant D (2006). Application of phylogenetic networks in evolutionary studies. Mol. Biol. Evol..

[27] Finn A (2016). Density Distribution of Pharyngeal Carriage of Meningococcus in Healthy Young Adults: New Approaches to Studying the Epidemiology of Colonization and Vaccine Indirect Effects. Pediatr. Infect. Dis. J..

[28] Lex A, Gehlenborg N, Strobelt H, Vuillemot R, Pfister H (2014). UpSet: Visualization of Intersecting Sets. IEEE Trans. Vis. Comput. Graph..

