# Supplementary material for: Outbreak strain characterisation and pharyngeal carriage detection following a protracted group B meningococcal outbreak in adolescents in South-West England
Source: Sci Rep. 2019 Jul 10;9:9990. doi: 10.1038/s41598-019-46483-3 (PMC6620271; doi:10.1038/s41598-019-46483-3)
Supplement: Supplementary file 1 — Dataset 1 [file 41598_2019_46483_MOESM1_ESM.docx]

**Manuscript Title:** Outbreak strain characterisation and pharyngeal carriage detection following a protracted group B meningococcal outbreak in adolescents in South-West England.

**Authors:** Stephen A. Clark, Jay Lucidarme, Georgina Angel, Aiswarya Lekshmi, Begonia Morales-Aza**,** Laura Willerton**,** Helen Campbell, Steve J. Gray, Shamez N. Ladhani, Mike Wade, Mary Ramsay, Julie Yates, Adam Finn, Ray Borrow

**Supplementary Table S1:** Meningococcal carriage among the 129 participants in a pharyngeal swabbing exercise. The columns in left-hand box contain the results of real-time PCR (*sodC* and *ctrA*) and sequencing of *PorA* directly from the swab. The columns in the right-hand box contain information on the isolates obtained through *in vitro* culture from the swabs. All swabs were numbered based on ascending *sodC* Ct value. Gray shading indicates positive meningococcal detection.

| **Swab No.** | ***sodC* Ct value** | ***ctrA* Ct value** | ***siaD_B_* Ct value** | **PorA genotype (direct)** | **Isolate ID** | **Phenotype** | **Genogroup** | **PorA** | **ST** | **clonal complex** | **Comments** |
| --- | --- | --- | --- | --- | --- | --- | --- | --- | --- | --- | --- |
| 1 | 27.43 | 27.10 | 27.54 | P1.22,14,36 | BRI011 | B;1;NT;14 | B | P1.22,14,36 | 2660 | ST-213 complex | n/a |
| 2 | 28.16 | ND | ND | P1.5,2,36-2 | BRI007 | NG;2a;5;2 | NG | P1.5,2,36-2 | 11 | ST-11 complex | Partial deletion of capsular locus |
| 3 | 29.98 | ND | ND | P1.18,25-44,38-1 | BRI002 | NG;15;NT;NT | NG | P1.18,25-44,38-1 | 823 | ST-198 complex | Capsular null locus |
| 4 | 30.89 | ND | ND | NP | ~ | ~ | ~ | ~ | ~ | ~ | ~ |
| 5 | 31.44 | 32.56 | 33.12 | P1.22,14,36 | BRI005 | B;4;NT;14 | B | P1.22,14,36 | 41 | ST-41/44 complex | n/a |
| 6 | 31.45 | 32.44 | 32.97 | P1.17-1,23,37 | BRI019 | B;NT;NT;NT | B | P1.17-1,23,37 | 1097 | ST-41/44 complex | n/a |
| 7 | 32.09 | 31.29 | ND | P1.7-2,13-9,35-1 | BRI006 | NG;NT;NT;13 | C | P1.7-2,13-9,35-1 | 278 | ST-35 complex | Internal stop codon in polysialyltransferase (*siaDc*) |
| 8 | 32.22 | 32.23 | 32.89 | P1.7,30-3,38 | BRI020 | B;NT;7;NT | B | P1.7,30-3,38 | 13745 | ST-213 complex | n/a |
| 9 | 32.42 | 33.79 | ND | P1.5-1,10-10,36-2 | BRI015 | NG;NT;5;NT | NG | P1.5-1,10-10,36-2 | 1655 | ST-23 complex | Capsule region A partially missing |
| 10 | 32.60 | 34.28 | ND | P1.5-1,2-2,36-2 | BRI018 | Y;NT;5;2 | Y | P1.5-1,2-2,36-2 | 23 | ST-23 complex | n/a |
| 11 | 32.93 | ND | ND | P1.18,25-44,38-1 | ~ | ~ | ~ | ~ | ~ | ~ | ~ |
| 12 | 33.26 | 32.51 | ND | P1.18-7,9,35-1 | BRI021 | NG;4;NT;9 | E | P1.18-7,9,35-1 | 254 | ST-254 complex | Serogroup E not part of routine phenotype testing |
| 13 | 33.30 | 33.48 | ND | P1.21-7,16,37-1 | BRI008 | NG;NT;NT;16 | E | P1.21-7,16,37-1 | 1157 | ST-1157 complex | Serogroup E not part of routine phenotype testing |
| 14 | 33.48 | 39.04 | ND | NP | BRI004 | NG;NT;5;2 | X | P1.5-1,2-5,36-2 | 5063 | Singleton | Serogroup X not part of routine phenotype testing |
| 15 | 33.55 | 33.44 | ND | P1.5,2,36-2 | BRI023 | NG;NT;5;2 | E | P1.5,2,36-2 | 60 | ST-60 complex | Serogroup E not part of routine phenotype testing |
| 16 | 33.68 | ND | ND | NP | ~ | ~ | ~ | ~ | ~ | ~ | ~ |
| 17 | 34.28 | 38.46 | ND | P1.5-1,2-59,36-2 | BRI022 | NG;4;5;2 | W | P1.5-1,-,36-2 | 910 | Singleton | n/a |
| 18 | 34.62 | 36.31 | ND | NP | ~ | ~ | ~ | ~ | ~ | ~ | ~ |
| 19 | 34.68 | 37.34 | ND | NP | ~ | ~ | ~ | ~ | ~ | ~ | ~ |
| 20 | 35.10 | ND | ND | NP | ~ | ~ | ~ | ~ | ~ | ~ | ~ |
| 21 | 35.14 | ND | ND | NP | ~ | ~ | ~ | ~ | ~ | ~ | ~ |
| 22 | 35.24 | ND | ND | NP | ~ | ~ | ~ | ~ | ~ | ~ | ~ |
| 23 | 35.30 | ND | ND | NP | ~ | ~ | ~ | ~ | ~ | ~ | ~ |
| 24 | 35.41 | 35.53 | ND | P1.5,2,36-2 | BRI016 | NG;NT;NT;2 | E | P1.5,2,36-2 | 13744 | ST-1157 complex | Serogroup E not part of routine phenotype testing |
| 25 | 35.42 | ND | ND | NP | ~ | ~ | ~ | ~ | ~ | ~ | ~ |
| 26 | 35.44 | 35.41 | 35.87 | P1.17-1,23,37 | BRI024 | NG;1;NT;NT | B | P1.17-1,23,37 | 1423 | ST-41/44 complex | Polysialyltransferase (*siaDb*) phase variable: off |
| 27 | 35.45 | ND | ND | NP | ~ | ~ | ~ | ~ | ~ | ~ | ~ |
| 28 | 35.50 | 38.31 | ND | NP | BRI009 | NG;NT;5;2 | E | P1.5,2,36-2 | 13744 | ST-1157 complex | Serogroup E not part of routine phenotype testing |
| 29 | 35.54 | ND | ND | NP | ~ | ~ | ~ | ~ | ~ | ~ | ~ |
| 30 | 35.61 | 35.96 | ND | P1.5,2,36-2 | BRI017 | W;2a;5;2 | W | P1.5,2,36-2 | 11 | ST-11 complex | n/a |
| 31 | 35.66 | ND | ND | NP | ~ | ~ | ~ | ~ | ~ | ~ | ~ |
| 32 | 35.86 | ND | ND | NP | ~ | ~ | ~ | ~ | ~ | ~ | ~ |
| 33 | 35.88 | ND | ND | NP | ~ | ~ | ~ | ~ | ~ | ~ | ~ |
| 34 | 35.96 | ND | ND | NP | ~ | ~ | ~ | ~ | ~ | ~ | ~ |
| 35 | 35.99 | ND | ND | NP | ~ | ~ | ~ | ~ | ~ | ~ | ~ |
| 36 | 36.08 | ND | ND | NP | ~ | ~ | ~ | ~ | ~ | ~ | ~ |
| 37 | 36.10 | ND | ND | NP | ~ | ~ | ~ | ~ | ~ | ~ | ~ |
| 38 | 36.17 | ND | ND | NP | ~ | ~ | ~ | ~ | ~ | ~ | ~ |
| 39 | 36.37 | ND | ND | NP | ~ | ~ | ~ | ~ | ~ | ~ | ~ |
| 40 | 36.48 | ND | ND | NP | ~ | ~ | ~ | ~ | ~ | ~ | ~ |
| 41 | 36.66 | ND | ND | NP | ~ | ~ | ~ | ~ | ~ | ~ | ~ |
| 42 | 36.81 | ND | ND | NP | ~ | ~ | ~ | ~ | ~ | ~ | ~ |
| 43 | 36.83 | ND | ND | NP | ~ | ~ | ~ | ~ | ~ | ~ | ~ |
| 44 | 36.83 | ND | ND | NP | ~ | ~ | ~ | ~ | ~ | ~ | ~ |
| 45 | 37.06 | ND | ND | NP | ~ | ~ | ~ | ~ | ~ | ~ | ~ |
| 46 | 37.09 | ND | ND | NP | ~ | ~ | ~ | ~ | ~ | ~ | ~ |
| 47 | 37.37 | ND | ND | NP | ~ | ~ | ~ | ~ | ~ | ~ | ~ |
| 48 | 37.39 | ND | ND | NP | ~ | ~ | ~ | ~ | ~ | ~ | ~ |
| 49 | 37.51 | ND | ND | NP | ~ | ~ | ~ | ~ | ~ | ~ | ~ |
| 50 | 37.59 | ND | ND | NP | ~ | ~ | ~ | ~ | ~ | ~ | ~ |
| 51 | 37.65 | ND | ND | NP | ~ | ~ | ~ | ~ | ~ | ~ | ~ |
| 52 | 37.66 | ND | ND | NP | ~ | ~ | ~ | ~ | ~ | ~ | ~ |
| 53 | 37.66 | ND | ND | NP | ~ | ~ | ~ | ~ | ~ | ~ | ~ |
| 54 | 37.72 | ND | ND | P1.18-7,9,35-1 | ~ | ~ | ~ | ~ | ~ | ~ | ~ |
| 55 | 37.79 | ND | ND | NP | ~ | ~ | ~ | ~ | ~ | ~ | ~ |
| 56 | 37.81 | ND | ND | NP | ~ | ~ | ~ | ~ | ~ | ~ | ~ |
| 57 | 37.87 | ND | ND | NP | ~ | ~ | ~ | ~ | ~ | ~ | ~ |
| 58 | 38.07 | 39.25 | ND | P1.5-1,10-1,36-2 | ~ | ~ | ~ | ~ | ~ | ~ | ~ |
| 59 | 38.09 | ND | ND | NP | ~ | ~ | ~ | ~ | ~ | ~ | ~ |
| 60 | 38.15 | ND | ND | NP | ~ | ~ | ~ | ~ | ~ | ~ | ~ |
| 61 | 38.18 | ND | ND | NP | ~ | ~ | ~ | ~ | ~ | ~ | ~ |
| 62 | 38.26 | ND | ND | NP | ~ | ~ | ~ | ~ | ~ | ~ | ~ |
| 63 | 38.31 | 38.81 | ND | P1.21-7,16,37-1 | ~ | ~ | ~ | ~ | ~ | ~ | ~ |
| 64 | 38.35 | ND | ND | NP | ~ | ~ | ~ | ~ | ~ | ~ | ~ |
| 65 | 38.57 | ND | ND | NP | ~ | ~ | ~ | ~ | ~ | ~ | ~ |
| 66 | 38.60 | ND | ND | NP | ~ | ~ | ~ | ~ | ~ | ~ | ~ |
| 67 | 38.70 | ND | ND | NP | ~ | ~ | ~ | ~ | ~ | ~ | ~ |
| 68 | 38.70 | ND | ND | NP | ~ | ~ | ~ | ~ | ~ | ~ | ~ |
| 69 | 38.72 | ND | ND | NP | ~ | ~ | ~ | ~ | ~ | ~ | ~ |
| 70 | 38.89 | ND | ND | NP | ~ | ~ | ~ | ~ | ~ | ~ | ~ |
| 71 | 38.92 | ND | ND | NP | ~ | ~ | ~ | ~ | ~ | ~ | ~ |
| 72 | 39.04 | 38.97 | ND | NP | ~ | ~ | ~ | ~ | ~ | ~ | ~ |
| 73 | 39.31 | ND | ND | NP | ~ | ~ | ~ | ~ | ~ | ~ | ~ |
| 74 | 39.32 | ND | ND | NP | ~ | ~ | ~ | ~ | ~ | ~ | ~ |
| 75 | 39.35 | ND | ND | NP | ~ | ~ | ~ | ~ | ~ | ~ | ~ |
| 76 | 39.44 | ND | ND | NP | ~ | ~ | ~ | ~ | ~ | ~ | ~ |
| 77 | 39.80 | ND | ND | NP | ~ | ~ | ~ | ~ | ~ | ~ | ~ |
| 78 | 39.83 | ND | ND | NP | ~ | ~ | ~ | ~ | ~ | ~ | ~ |
| 79 | 40.10 | ND | ND | NP | ~ | ~ | ~ | ~ | ~ | ~ | ~ |
| 80 | 40.39 | ND | ND | NP | ~ | ~ | ~ | ~ | ~ | ~ | ~ |
| 81 | 40.62 | ND | ND | NP | ~ | ~ | ~ | ~ | ~ | ~ | ~ |
| 82 | 40.71 | ND | ND | NP | ~ | ~ | ~ | ~ | ~ | ~ | ~ |
| 83 | 40.88 | ND | ND | NP | ~ | ~ | ~ | ~ | ~ | ~ | ~ |
| 84 | 41.08 | ND | ND | NP | ~ | ~ | ~ | ~ | ~ | ~ | ~ |
| 85 | 41.24 | ND | ND | NP | ~ | ~ | ~ | ~ | ~ | ~ | ~ |
| 86 | 41.86 | ND | ND | NP | ~ | ~ | ~ | ~ | ~ | ~ | ~ |
| 87 | 41.97 | ND | ND | NP | ~ | ~ | ~ | ~ | ~ | ~ | ~ |
| 88 | 42.07 | ND | ND | NP | ~ | ~ | ~ | ~ | ~ | ~ | ~ |
| 89 | 42.48 | 38.69 | ND | NP | ~ | ~ | ~ | ~ | ~ | ~ | ~ |
| 90 | 43.75 | ND | ND | NP | ~ | ~ | ~ | ~ | ~ | ~ | ~ |
| 91 | 44.70 | ND | ND | NP | ~ | ~ | ~ | ~ | ~ | ~ | ~ |
| 92 | 44.89 | ND | ND | NP | ~ | ~ | ~ | ~ | ~ | ~ | ~ |
| 93 | 44.92 | ND | ND | NP | ~ | ~ | ~ | ~ | ~ | ~ | ~ |
| 94 | 45.03 | ND | ND | NP | ~ | ~ | ~ | ~ | ~ | ~ | ~ |
| 95 | 45.12 | ND | ND | NP | ~ | ~ | ~ | ~ | ~ | ~ | ~ |
| 96 | 46.27 | ND | ND | NP | ~ | ~ | ~ | ~ | ~ | ~ | ~ |
| 97 | ND | ND | ND | P1.17,9,35-1 | ~ | ~ | ~ | ~ | ~ | ~ | ~ |
| 98 | ND | 36.70 | 38.51 | P1.22,14,36 | ~ | ~ | ~ | ~ | ~ | ~ | ~ |
| 99 | ND | ND | ND | NP | ~ | ~ | ~ | ~ | ~ | ~ | ~ |
| 100 | ND | ND | ND | NP | ~ | ~ | ~ | ~ | ~ | ~ | ~ |
| 101 | ND | ND | ND | NP | ~ | ~ | ~ | ~ | ~ | ~ | ~ |
| 102 | ND | ND | ND | NP | ~ | ~ | ~ | ~ | ~ | ~ | ~ |
| 103 | ND | ND | ND | NP | ~ | ~ | ~ | ~ | ~ | ~ | ~ |
| 104 | ND | ND | ND | NP | ~ | ~ | ~ | ~ | ~ | ~ | ~ |
| 105 | ND | ND | ND | NP | ~ | ~ | ~ | ~ | ~ | ~ | ~ |
| 106 | ND | ND | ND | NP | ~ | ~ | ~ | ~ | ~ | ~ | ~ |
| 107 | ND | ND | ND | NP | ~ | ~ | ~ | ~ | ~ | ~ | ~ |
| 108 | ND | ND | ND | NP | ~ | ~ | ~ | ~ | ~ | ~ | ~ |
| 109 | ND | ND | ND | NP | ~ | ~ | ~ | ~ | ~ | ~ | ~ |
| 110 | ND | ND | ND | NP | ~ | ~ | ~ | ~ | ~ | ~ | ~ |
| 111 | ND | ND | ND | NP | ~ | ~ | ~ | ~ | ~ | ~ | ~ |
| 112 | ND | ND | ND | NP | ~ | ~ | ~ | ~ | ~ | ~ | ~ |
| 113 | ND | ND | ND | NP | ~ | ~ | ~ | ~ | ~ | ~ | ~ |
| 114 | ND | ND | ND | NP | ~ | ~ | ~ | ~ | ~ | ~ | ~ |
| 115 | ND | ND | ND | NP | ~ | ~ | ~ | ~ | ~ | ~ | ~ |
| 116 | ND | ND | ND | NP | ~ | ~ | ~ | ~ | ~ | ~ | ~ |
| 117 | ND | ND | ND | NP | ~ | ~ | ~ | ~ | ~ | ~ | ~ |
| 118 | ND | ND | ND | NP | ~ | ~ | ~ | ~ | ~ | ~ | ~ |
| 119 | ND | ND | ND | NP | ~ | ~ | ~ | ~ | ~ | ~ | ~ |
| 120 | ND | ND | ND | NP | ~ | ~ | ~ | ~ | ~ | ~ | ~ |
| 121 | ND | ND | ND | NP | ~ | ~ | ~ | ~ | ~ | ~ | ~ |
| 122 | ND | ND | ND | NP | ~ | ~ | ~ | ~ | ~ | ~ | ~ |
| 123 | ND | ND | ND | NP | ~ | ~ | ~ | ~ | ~ | ~ | ~ |
| 124 | ND | ND | ND | NP | ~ | ~ | ~ | ~ | ~ | ~ | ~ |
| 125 | ND | ND | ND | NP | ~ | ~ | ~ | ~ | ~ | ~ | ~ |
| 126 | ND | ND | ND | NP | ~ | ~ | ~ | ~ | ~ | ~ | ~ |
| 127 | ND | ND | ND | NP | ~ | ~ | ~ | ~ | ~ | ~ | ~ |
| 128 | ND | ND | ND | NP | ~ | ~ | ~ | ~ | ~ | ~ | ~ |
| 129 | ND | ND | ND | NP | ~ | ~ | ~ | ~ | ~ | ~ | ~ |

~ isolate not obtained

ND: Not detected

NP: No sequencable PCR product obtained

**Supplementary Table S2:** fHbp sequencing results direct from swab (left-hand box) alongside fHbp data from corresponding isolates (right-hand box, where applicable). Discordant results are highlighted in bold text. ‘NP’ indicates no PCR product was obtained.

| **Swab No.** | **fHbp allele (from swab)** | **fHbp peptide (from swab)** | **Isolate ID** | **fHbp allele (from isolate)** | **fHbp peptide (from isolate)** |
| --- | --- | --- | --- | --- | --- |
| 1 | 65 | 45 | BRI011 | 65 | 45 |
| 2 | 22 | 22 | BRI007 | 22 | 22 |
| 3 | 66 | 4 | BRI002 | 66 | 4 |
| 4 | 928 | 762 | ~ | ~ | ~ |
| 5 | **288** | **235** | BRI005 | **4** | **4** |
| 6 | **522** | **447** | BRI019 | **19** | **19** |
| 7 | 24 | 24 | BRI006 | 24 | 24 |
| 8 | **1532** | **401** | BRI020 | **65** | **45** |
| 9 | **778** | **499** | BRI015 | **25** | **25** |
| 10 | **110** | **110** | BRI018 | **25** | **25** |
| 11 | NP | NP | ~ | ~ | ~ |
| 12 | 13 | 13 | BRI021 | 13 | 13 |
| 13 | 68 | 13 | BRI008 | 68 | 13 |
| 14 | 108/34* | 108/12* | BRI004 | 34 | 12 |
| 15 | 386/13* | 312/13* | BRI023 | 13 | 13 |
| 16 | 108 | 108 | ~ | ~ | ~ |
| 17 | **47** | **61** | BRI022 | 24 | 24 |
| 18 | 928 | 762 | ~ | ~ | ~ |
| 19 | 108 | 108 | ~ | ~ | ~ |
| 20 | 30 | 30 | ~ | ~ | ~ |
| 21 | 15 | 15 | ~ | ~ | ~ |
| 22 | 323 | 276 | ~ | ~ | ~ |
| 23 | NP | NP | ~ | ~ | ~ |
| 24 | **778** | **499** | BRI016 | **13** | **13** |
| 25 | NP | NP | ~ | ~ | ~ |
| 26 | **33** | **31** | BRI024 | **19** | **19** |
| 27 | NP | NP | ~ | ~ | ~ |
| 28 | 13 | 13 | BRI009 | 13 | 13 |
| 29 | 1161 | 925 | ~ | ~ | ~ |
| 30 | **546/13*** | **456/13*** | BRI017 | **22** | **22** |
| 31 | 1173 | 933 | ~ | ~ | ~ |
| 32 | 881 | 722 | ~ | ~ | ~ |
| 33 | 673 | 570 | ~ | ~ | ~ |
| 34 | 1035 | 829 | ~ | ~ | ~ |
| 35 | NP | NP | ~ | ~ | ~ |
| 36 | 98 | 90 | ~ | ~ | ~ |
| 37 | 110 | 110 | ~ | ~ | ~ |
| 38 | 505 | 430 | ~ | ~ | ~ |
| 39 | 33 | 31 | ~ | ~ | ~ |
| 40 | 47 | 61 | ~ | ~ | ~ |
| 41 | 47 | 61 | ~ | ~ | ~ |
| 42 | 1542 | 1179 | ~ | ~ | ~ |
| 43 | 1537 | 1091 | ~ | ~ | ~ |
| 44 | 1538 | 1178 | ~ | ~ | ~ |
| 45 | 522 | 447 | ~ | ~ | ~ |
| 46 | 30 | 30 | ~ | ~ | ~ |
| 47 | 928 | 762 | ~ | ~ | ~ |
| 48 | Mixed | Mixed | ~ | ~ | ~ |
| 49 | 110 | 110 | ~ | ~ | ~ |
| 50 | 108 | 108 | ~ | ~ | ~ |
| 51 | NP | NP | ~ | ~ | ~ |
| 52 | NP | NP | ~ | ~ | ~ |
| 53 | 505 | 430 | ~ | ~ | ~ |
| 54 | 110 | 110 | ~ | ~ | ~ |
| 55 | NP | NP | ~ | ~ | ~ |
| 56 | 1282 | 1008 | ~ | ~ | ~ |
| 57 | NP | NP | ~ | ~ | ~ |
| 58 | 1539 | n/a** | ~ | ~ | ~ |
| 59 | 110 | 110 | ~ | ~ | ~ |
| 60 | 108 | 108 | ~ | ~ | ~ |
| 61 | 108 | 108 | ~ | ~ | ~ |
| 62 | 1529 | 1172 | ~ | ~ | ~ |
| 63 | 110 | 110 | ~ | ~ | ~ |
| 64 | 108 | 108 | ~ | ~ | ~ |
| 65 | NP | NP | ~ | ~ | ~ |
| 66 | 1431 | 218 | ~ | ~ | ~ |
| 67 | NP | NP | ~ | ~ | ~ |
| 68 | 928 | 762 | ~ | ~ | ~ |
| 69 | 1541 | 312 | ~ | ~ | ~ |
| 70 | NP | NP | ~ | ~ | ~ |
| 71 | 33 | 31 | ~ | ~ | ~ |
| 72 | 165 | 156 | ~ | ~ | ~ |
| 73 | 277 | 226 | ~ | ~ | ~ |
| 74 | 928 | 762 | ~ | ~ | ~ |
| 75 | 1530 | 312 | ~ | ~ | ~ |
| 76 | NP | NP | ~ | ~ | ~ |
| 77 | 1528 | 1171 | ~ | ~ | ~ |
| 78 | NP | NP | ~ | ~ | ~ |
| 79 | 277 | 226 | ~ | ~ | ~ |
| 80 | 47 | 61 | ~ | ~ | ~ |
| 81 | 971 | 456 | ~ | ~ | ~ |
| 82 | 1541 | 312 | ~ | ~ | ~ |
| 83 | 47 | 61 | ~ | ~ | ~ |
| 84 | 108 | 108 | ~ | ~ | ~ |
| 85 | NP | NP | ~ | ~ | ~ |
| 86 | NP | NP | ~ | ~ | ~ |
| 87 | NP | NP | ~ | ~ | ~ |
| 88 | 928 | 762 | ~ | ~ | ~ |
| 89 | 368 | 312 | ~ | ~ | ~ |
| 90 | NP | NP | ~ | ~ | ~ |
| 91 | 368 | 312 | ~ | ~ | ~ |
| 92 | 108 | 108 | ~ | ~ | ~ |
| 93 | 350 | 297 | ~ | ~ | ~ |
| 94 | NP | NP | ~ | ~ | ~ |
| 95 | 288 | 235 | ~ | ~ | ~ |
| 96 | 538 | 464 | ~ | ~ | ~ |
| 97 | NP | NP | ~ | ~ | ~ |
| 98 | 33 | 31 | ~ | ~ | ~ |
| 99 | NP | NP | ~ | ~ | ~ |
| 100 | 68 | 13 | ~ | ~ | ~ |
| 101 | NP | NP | ~ | ~ | ~ |
| 102 | 47 | 61 | ~ | ~ | ~ |
| 103 | 673 | 570 | ~ | ~ | ~ |
| 104 | NP | NP | ~ | ~ | ~ |
| 105 | NP | NP | ~ | ~ | ~ |
| 106 | NP | NP | ~ | ~ | ~ |
| 107 | 824 | 677 | ~ | ~ | ~ |
| 108 | 1531 | 1174 | ~ | ~ | ~ |
| 109 | 302 | 245 | ~ | ~ | ~ |
| 110 | 110 | 110 | ~ | ~ | ~ |
| 111 | 108 | 108 | ~ | ~ | ~ |
| 112 | 505 | 430 | ~ | ~ | ~ |
| 113 | 56 | 69 | ~ | ~ | ~ |
| 114 | 1503 | 1150 | ~ | ~ | ~ |
| 115 | 1534 | 1176 | ~ | ~ | ~ |
| 116 | NP | NP | ~ | ~ | ~ |
| 117 | 1535 | n/a** | ~ | ~ | ~ |
| 118 | 1536 | 1177 | ~ | ~ | ~ |
| 119 | 546 | 456 | ~ | ~ | ~ |
| 120 | NP | NP | ~ | ~ | ~ |
| 121 | NP | NP | ~ | ~ | ~ |
| 122 | 6 | 6 | ~ | ~ | ~ |
| 123 | 1540 | 1173 | ~ | ~ | ~ |
| 124 | 15 | 15 | ~ | ~ | ~ |
| 125 | 566 | 490 | ~ | ~ | ~ |
| 126 | 47 | 61 | ~ | ~ | ~ |
| 127 | 990 | 799 | ~ | ~ | ~ |
| 128 | 673 | 570 | ~ | ~ | ~ |
| 129 | 110 | 110 | ~ | ~ | ~ |
| * multiple alleles discerned among traces | | | |  |  |
| ** No peptide ID. Internal stop codon | | | |  |  |
| ~ isolate not obtained  NP: No sequencable PCR product obtained | | |  |  |  |
